# Parent Psychopathology and Neurocognitive Functioning in Children With ADHD

**DOI:** 10.1177/1087054717718262

**Published:** 2017-07-08

**Authors:** Sharifah Shameem Agha, Stanley Zammit, Anita Thapar, Kate Langley

**Affiliations:** 1 Cardiff University, UK; 2 Cwm Taf University Health Board, UK; 3 University of Bristol, UK

**Keywords:** ADHD, neurocognitive functioning, parent ADHD, parent depression

## Abstract

**Objective:** The objective of this study was to examine the association between parent mental health (ADHD and depression) and offspring performance on neurocognitive tasks in children with ADHD. **Method:** The clinical sample consisted of 570 children (85% males, mean age: 10.77 years) with ADHD who completed neurocognitive tasks measuring working memory, attention set-shifting, and motivational deficits. Questionnaire measures were used to assess ADHD and depression symptom presence in parents. **Results:** Controlling for ADHD severity, children of parents with ADHD had poorer working memory (*B* = −0.25, 95% confidence interval [CI] [−0.45, −0.07], *p* = .01) and increased errors on the extra dimensional shift stage of the set-shifting task (*B* = 0.26 95% CI [0.02, 0.50], *p* = .04). Parent depression was not associated with offspring performance on any of the assessed neurocognitive tasks. **Conclusion:** Children with ADHD who have a parent with ADHD symptom presence are a subgroup of children who may have additional neurocognitive impairments that have potential implications when implementing interventions that target cognition and learning.

## Introduction

ADHD is a common childhood neurodevelopmental disorder characterized by impairing levels of inattention and hyperactivity-impulsiveness. ADHD is a genetically influenced and highly familial disorder, although noninherited factors also contribute (Thapar, Cooper, Eyre, & Langley, 2013). Psychopathology rates have been found to be high in parents of children with ADHD (Barkley, 1998; Johnston & Mash, 2001); 25% to 50% of children with ADHD are reported to have a parent with ADHD (Biederman et al., 1992; Chronis et al., 2003; Vidair et al., 2011), and higher rates of depression also are found among parents of children with ADHD compared with parents of unaffected children (Chronis et al., 2003; Faraone & Biederman, 1997; Margari et al., 2013).

Previous research, including our own, has shown that parental ADHD is associated with a more severe clinical presentation of the same disorder in offspring, including higher ADHD symptom severity and elevated rates of comorbid conduct symptoms and disorder (Agha, Zammit, Thapar, & Langley, 2013; López Seco et al., 2015; Segenreich et al., 2014). There is also evidence that parental depression is associated with a more severe clinical presentation and greater impairment in children with ADHD (Chronis et al., 2003; Humphreys, Mehta, & Lee, 2012; Pressman et al., 2006) and is associated with later development of conduct disorder (CD) symptoms in children (Agha, Zammit, Thapar, & Langley, 2017).

ADHD is characterized by neurocognitive deficits as well as by its core clinical features (Willcutt, Doyle, Nigg, Faraone, & Pennington, 2005). Children with ADHD manifest deficits in various neurocognitive domains including executive function (Seidman, 2006; Willcutt et al., 2005) and delay aversion (Sonuga-Barke, 2002). Just as with the complex clinical nature of ADHD, there is heterogeneity in neurocognitive performance among children with ADHD (Doyle, 2006; Nigg, Blaskey, Stawicki, & Sachek, 2004). A number of studies have demonstrated that variability in neurocognitive performance among children with ADHD is associated with comorbidity and worse outcomes in adolescence and adulthood (Doyle, 2006; Nikolas & Nigg, 2014; van Lieshout, Luman, Buitelaar, Rommelse, & Oosterlaan, 2013; van Lieshout et al., 2017). Thus, such deficits provide an alternative index of ADHD severity. Many of the studies looking at associations between parental psychopathology and offspring ADHD phenotypic characteristics utilize subjective reports of clinical severity in the child that in many cases have been provided by the parent. It is possible, therefore, that the parents’ own mental state and psychopathology may influence their reporting of the child’s behavior. Neurocognitive measures provide a more objective and nonbehavioral measure of impairment in children with ADHD compared with subjective parent reports or reported symptoms. Therefore, investigating the relationship between parental psychopathology and neurocognitive variability in ADHD provides an additional opportunity to empirically assess the relevance of parental mental health to the clinical severity of offspring ADHD.

The few studies to date that have undertaken this type of investigation have somewhat mixed findings. A number of studies report that children with a parent or first-degree relative with ADHD show poorer performance on inhibition, set-shifting, and verbal cognitive ability (Crosbie & Schachar, 2001; Seidman et al., 1995; Seidman, Biederman, Faraone, Weber, & Ouellette, 1997; Thissen, Rommelse, Altink, Oosterlaan, & Buitelaar, 2014) while others (Goos, Crosbie, Payne, & Schachar, 2009) find no such associations. To our knowledge, only one small, pilot study to date has investigated the association between parental depression and neurocognitive profiles in children with ADHD (Park et al., 2014). These authors found significantly poorer performance on an inhibition task and a visual attention task, although no differences on a range of other neurocognitive domains in those children with a parent who had a history of mood disorder.

Given the somewhat inconsistent as well as limited body of evidence to date, this study will explore the associations between parental ADHD and depression and neurocognitive performance in a sample of children with a clinical diagnosis of ADHD. General cognitive ability and three domains of neurocognition, previously shown to be associated with ADHD (Willcutt et al., 2005), were chosen for examination: working memory, set-shifting ability, and motivational decision making. Findings may help add potential insight into how parent ADHD is related to some aspects of offspring neurocognitive performance, which is another important manifestation of the ADHD phenotype.

## Method

### Sample

This sample of children with ADHD and their parents was recruited from child and adolescent psychiatry and pediatric services in the United Kingdom and has been described previously (Langley et al., 2011). All children referred had a clinical diagnosis of ADHD or were currently being assessed for a diagnosis and IQ was not an exclusion criterion for this study. Each child had to be living with at least one biological parent. As the study began in 2007, *Diagnostic and Statistical Manual of Mental Disorders* (4th ed.; *DSM-IV*; American Psychiatric Association, 1994) diagnostic criteria were utilized, and all children met research diagnostic criteria for ADHD according to *DSM-IV*, and *DSM-III-R* (American Psychiatric Association, 1987) (where teacher reports of pervasiveness were not available). Following the publication of *DSM-5* (American Psychiatric Association, 2013), two child psychiatrists reviewed the research interviews according to these criteria. All children meeting *DSM-IV* criteria had also met *DSM-5* criteria for ADHD. For the present analyses, where more than one child from the same family (*n* = 46) participated in the study, only one child (the oldest) was included in analysis. Parents and children gave informed written consent and assent before taking part in the study. Ethical approval for the study was obtained from the Wales Multicentre Research Ethics Committee.

### Measures

Parent psychopathology was assessed using questionnaire measures. Biological mothers and fathers completed a questionnaire regarding ADHD symptoms in themselves at age 7 to 11 years (childhood) and in the last 6 months (current), using an 18-item checklist of *DSM-5* ADHD symptoms (see Agha et al., 2013, for further details). Total scores were generated separately for childhood and current symptoms. Positive ADHD status was assigned if symptom criteria were met for a *DSM-5* ADHD diagnosis (a minimum of six inattentive or hyperactive/impulsive symptoms in childhood and at least five inattentive or hyperactive/impulsive symptoms at present). Parent ADHD symptom presence was defined as either mother or father having a positive ADHD status. Cronbach’s alpha reliability for parent ADHD measures ranged from 0.91 to 0.94.

To assess parent depression, biological mothers and fathers completed the Hospital Anxiety and Depression Scale (HADS; Zigmond & Snaith, 1983). As in previous validation studies, a cutoff score of 11 or higher was used to indicate the presence of a mood disorder based on seven depression items from the HADS (Bjelland, Dahl, Haug, & Neckelmann, 2002; Snaith, 2003). Parental depression was defined as either a mother or father with a mood disorder based on the cutoff score from the HADS.

Child psychopathology was assessed using the Child and Adolescent Psychiatric Assessment (CAPA), a semi-structured research diagnostic interview (Angold, Costello, & Erkanli, 1999). The parent version of the CAPA was used to assess the child’s clinical symptoms of ADHD, oppositional defiant disorder (ODD), CD, tic disorder, anxiety disorder and depression and associated impairment. The child version of the CAPA (this does not include an ADHD section) was additionally used for children aged 12 years and above. To assess pervasiveness of ADHD symptoms across settings, reports from schools were obtained using the Child ADHD Teacher Telephone Interview (ChATTI; Holmes et al., 2004), the Conner’s Teacher Rating Scale (Conners, Sitarenios, Parker, & Epstein, 1998), or DuPaul teacher rating scales (DuPaul, 1981). All interviews were administered by trained psychologists supervised weekly by a child psychiatrist (A.T.) and a psychologist (K.L.). Total symptom scores and diagnoses for the current analysis were generated from the CAPA according to *DSM-5* criteria. Individual symptoms of CD and ODD were counted as present when endorsed by either the parent or child and summed to calculate severity scores.

#### Neurocognitive measures

All cognitive assessments were performed by trained psychologists. Children were requested to be off their stimulant medication 24 hr prior to testing. Cognitive ability was assessed using the Wechsler Intelligence Scale for Children IV (WISC-IV) where a measure of full scale IQ was obtained (Wechsler, 2003). The Digit Span subtest is a measure of verbal working memory. Children are verbally given sequences of numbers and asked to repeat them, either as heard or in reverse order. This task has been used in previous research to assess working memory in children with ADHD (Gau & Shang, 2010).

The Intra-Extra Dimensional Set Shift (IED) task is taken from the Cambridge Neurocognitive Test Automated Battery (CANTAB), a computerized battery of nonverbal visually presented neurocognitive tests (Cambridge Cognition, 1996). It is a computerized analogue version of the Wisconsin Card Sorting test and largely used as an executive functioning measure of visual discrimination, set-shifting, and attention flexibility. Participants are presented with two types of dimensions/shapes (simple and compound) and are asked to choose a pattern they think is correct. Feedback teaches the participant which is the correct rule, and they need to follow it until the rule changes again. There are a total of nine stages, where at each stage the participant has to learn the visual discrimination. Progress on to the next stage is dependent on a criterion of six consecutive correct responses (Downes et al., 1989; Syngelaki, Moore, Savage, Fairchild, & Van Goozen, 2009). There are two key stages here: (a) Stage 6—intra dimensional shift (ID), which requires participants to maintain attention to a previously relevant dimension, and (b) Stage 8—extra dimensional shift (ED) where participants need to shift their attention to a previously irrelevant dimension. The outcome measure is the total number of errors made throughout the task (adjusted for any stage that was not attempted) and the number of errors made in the ED shift stage (Stage 8).

The Cambridge Gambling Task (CGT), also part of the Cambridge Neurocognitive Battery (CANTAB) assesses decision making and delay aversion (Rogers et al., 1999). On each trial, participants are presented with different ratios of 10 red and blue boxes in which a yellow token is hidden. Participants must guess whether the yellow token is concealed behind a red or blue square. The participants start with a number of points displayed on the screen and must then select/bet a proportion of these points, displayed in either ascending or descending order, to indicate their confidence of their chosen color. The aim is to accumulate as many points as possible. The outcome measures used were *quality of decision making* which looks at the proportion of trials where the majority color was chosen (a higher score is favorable), *delay aversion* which is difference in percentage bets on the descending versus ascending trials (higher scores indicate impulsivity and intolerance of waiting), *risk adjustment* that is the rate at which subjects increase the bet proportion in response to more favorable ratios (low scores are unfavorable), and *risk taking* that is the mean proportion of points bet on trials where the most likely outcome was chosen (DeVito et al., 2008; Groen, Gaastra, Lewis-Evans, & Tucha, 2013).

Information on demographics and family background was obtained from each family using a self-report parent questionnaire. Social class status was classified according to the occupation of the main family wage earner, using the U.K. Standard Occupational Classification 2000 (Office for National Statistics, 2000). Families were then categorized as having a lower social economic status or not, with lower socioeconomic status defined as being in unskilled employment/unemployment. Parent education was based on the variable of low educational attainment, that is, having left school without qualifications (General Certificate of Secondary Education (GCSE) or equivalent) or otherwise. Information on ADHD medication was also collected, and children were classified according to whether or not they had a current prescription for ADHD medication.

### Analysis

Linear regressions were used to examine association between predictors (parent ADHD/parent depression symptom presence) and outcomes (child scores on the neurocognitive tasks). All neurocognitive outcome scores were standardized for ease of interpretation and comparison across different tasks. Estimates were then further adjusted for child age, low social class, and low parent education to test whether associations found were explained by parent level of education (as a proxy measure of parent IQ) and by social class status. Child IQ was included as a covariate in the subsequent model but not for the analysis looking at Digit Span as it is one of the subtests used to assess full scale IQ. In the final model, child ADHD and CD severity was included as a covariate to determine whether associations between parent psychopathology and child neurocognitive performance were independent of child psychopathology. Post estimation tests identified two outliers within the digit span scores. As these outliers had higher than average leverage and residual points, it was decided that these two individuals would be excluded from the analyses. All analyses were performed using STATA (version13). The analyses in this study were exploratory without any correction for multiple testing.

## Results

### Sample Description

This sample consisted of 568 children aged 6 to 18 years, 480 (85%) males with a mean age of 10.77 years (*SD* = 3.01). All children had a clinical and research diagnosis of ADHD (International Classification of Diseases (ICD-10) Hyperkinetic Disorder or *DSM* ADHD). Rates of ADHD subtypes and comorbidities are reported in more detail in Agha et al. (2013).

ADHD and depression symptoms were available from completed questionnaire for 546 mothers and 280 fathers. The sample consisted of many single parent families; 58.7% (mostly mothers) of which many had fathers without questionnaire data available (50.9 %). Overall, 33% (*n* = 186) of children in the sample had a parent meeting symptom criteria for *DSM-5* ADHD. There were only a few children where both mothers and fathers had ADHD in the same family (1.9%, *n* = 11), and there were no significant correlations between mother and father ADHD total symptom scores (for either current or childhood symptoms). Looking at parent depression, 23.8% (*n* = 133) of children had a parent who met the cut point for depression based on the HADS. Only 1.4% (*n* = 8) of children had both parents meeting study criteria for depression.

Child age, gender, ADHD medication status of the child, and parent education level did not differ between those with and without a parent with ADHD or depression symptom presence. We found that 62% of families with a parent with ADHD were classified as being of low social class compared with 50% of families without an ADHD parent (χ^2^ = 6.69 (1), *p* = .01). There were no significant differences in terms of social class for parents with and without depression, but there was weak evidence that families with a parent with depression were more likely to be classified in the lower social class compared with families without a parent with depression (61% vs. 52%; χ^2^ = 3.04 (1), *p* = .081). There were 452 (84%) children who were not on stimulant medication 24 hr before cognitive testing and 84 (16%) who were on medication at time of assessment. Medication taken at the time of assessment was not significantly associated with any cognitive test scores.

Associations between child clinical symptoms and neurocognitive tasks were assessed next. ADHD symptom severity was found to be positively correlated with errors in the set-shifting task and negatively correlated with quality of decision making and risk-taking scores, whereas CD symptoms were negatively correlated with IQ and digit span. However, these correlation coefficients were small, ranging between *r* = .17 and .26. Table 1 provides a description of the sample in terms of neurocognitive task measures. As anticipated and previously observed in other studies, participants in this sample performed more poorly on all tasks than those in published normal population samples (DeVito et al., 2008; Flanagan & Kaufman, 2004; Gau & Shang, 2010). In the set-shifting task, the mean number of stages passed was 7.90 (*SD* = 0.96). Most children in this sample had completed the intra dimensional shift stage (ID—Stage 6), but just less than half the sample (49%) couldn’t complete the extra dimensional (ED) shift stage of the task (Stage 8). While not matched to the sample in this study, normative data on the set-shifting tasks reported mean number of stages passed ranging from 8.60 (*SD* = 0.97) to 8.68 (*SD* = 0.85) for children between ages 8 and 19 years (Strauss, Sherman, & Spreen, 2006).

**Table 1.** Associations Between Parent ADHD (Mother/Father) and Child Neurocognitive Performance.

Neurocognitive outcome	*n*	Total sample (*n* = 568)	Parent ADHD		Unadjusted model^a^		
			No (*n* = 380)	Yes (*n* = 186)	*B*	(95% CI)	*p*
		*M* (*SD*)	*M* (*SD*)	*M* (*SD*)			
Working memory							
Digit Span	520	7.14 (2.72)	7.35 (2.81)	6.66 (2.81)	−0.25	[−0.42, −0.07]	.01
Cognitive ability							
IQ	521	82.30 (13.54)	82.62 (13.59)	81.51 (13.43)	−0.08	[−0.26, 0.10]	.38
Attention set-shifting							
Total errors—set-shifting task	278	44.29 (21.22)	43.01 (21.45)	46.53 (20.22)	0.17	[−0.08, 0.41]	.18
ED shift errors	278	16.57 (10.50)	16.93 (10.14)	19.56 (9.63)	0.26	[0.02, 0.50]	.04
Motivational deficits							
Quality of decision making	296	0.76 (0.19)	0.77 (0.20)	0.76 (0.18)	−0.04	[−0.28, 0.20]	.75
Delay aversion	207	0.57 (0.20)	0.57 (0.21)	0.57 (0.20)	0.01	[−0.28, 0.29]	.97
Risk taking	294	0.54 (0.17)	0.54 (0.17)	0.55 (0.16)	−0.05	[−0.19, 0.29]	.69
Risk adjustment	294	0.31 (0.89)	0.31 (0.93)	0.32 (0.80)	−0.01	[−0.23, 0.25]	.96

*Note.* CI = confidence interval; ED = extra dimensional shift stage (Stage 8).

a Neurocognitive outcome variables in the unadjusted model are based on standardized scores.

### Parent Psychopathology and Offspring Neurocognitive Task Performance

These results are presented in Table 1. Parent ADHD symptom presence was found to be associated with lower offspring scores on the Digit Span subtest (*B* = −0.25, 95% CI [−0.45, −0.07], *p* = .01) and higher scores on total number of errors made in the ED shift stage (*B* = 0.26 95% CI [0.02, 0.50], *p* = .04). We found that only 44% of children with a parent with ADHD completed Stage 8/9 of the set-shifting task compared with 55% in the group of children without a parent with ADHD (χ^2^ = 3.32 (1), *p* = .07).

Parent ADHD symptom presence was not associated with total errors made on the set-shifting task and any of the measures from the gambling task (delay aversion, quality of decision making, risk adjustment, and risk-taking behavior; see Table 1). The effect sizes and pattern of results remained similar after adjusting for the covariates which indicates that the associations are independent of and not explained by child ADHD severity (see Table 2).

**Table 2.** Associations Between Parent ADHD (Mother/Father) and Child Neurocognitive Performance Adjusting for Covariates (Low Parent Education Status, Low Social Class, Child Age, Child IQ, and ADHD and Conduct Symptom Severity).

Neurocognitive outcome	Model 1 (Low parent education, low social class, & age)			Model 2 + (IQ)			Model 3 +(ADHD & CD severity)		
	*B*	(95% CI)	*p*	*B*	(95% CI)	*p*	*B*	(95% CI)	*p*
Working memory									
Digit Span^a^	−0.24	[−0.43, −0.05]	.01				−0.24	[−0.43, −0.04]	.02
Cognitive ability									
IQ	−0.05	[−0.24, 0.14]	.62				−0.02	[−0.21, 0.17]	.82
Attention set-shifting									
Total errors—set-shifting task	0.20	[−0.05, 0.45]	.12	0.18	[−0.08, 0.43]	0.17	0.21	[−0.05, 0.47]	.11
ED shift errors	0.30	[0.03, 0.55]	.03	0.27	[0.004, 0.53]	0.05	0.29	[0.02, 0.55]	.03
Motivational deficits									
Quality of decision making	−0.03	[−0.27, 0.21]	.80	−0.03	[−0.28, 0.22]	0.82	−0.04	[−0.29, 0.22]	.79
Delay aversion	0.03	[−0.27, 0.32]	.87	0.03	[−0.27, 0.33]	0.83	0.04	[−0.27, 0.35]	.80
Risk taking	0.06	[−0.18, 0.30]	.61	0.08	[−0.16, 0.32]	0.51	0.07	[−0.18, 0.32]	.56
Risk adjustment	0.03	[−0.23, 0.28]	.83	0.03	[−0.23, 0.29]	0.81	0.06	[−0.20 0.32]	.66

*Note.* Neurocognitive outcome variables in all models are based on standardized scores. CD = conduct disorder; CI = confidence interval; ED = extra dimensional shift stage (Stage 8).

a Digit Span subtest is included as part of the full scale IQ estimate; therefore, this was not controlled for.

We did not observe associations between parent depression symptom presence and any of the offspring neurocognitive outcome scores, apart from weak evidence of association with the delay aversion score (*B* = 0.29, 95% CI [−0.02, 0.60], *p* = .07; see Table 3). After adjustment for covariates, the pattern of associations did not change (see Table 4). In the parent depression groups, 42% of offspring with a parent with depression completed Stage 8/9 compared with 53% of children whose parent did not have depression (χ^2^ = 2.31 (1), *p* = .13).

**Table 3.** Associations Between Parent Depression (Mother/Father) and Child Neurocognitive Performance.

Neurocognitive outcome	*n*	Parent depression		Unadjusted model^a^		
		No (*n* = 425)	Yes (*n* = 133)	*B*	(95% CI)	*p*
		*M* (*SD*)	*M* (*SD*)			
Working memory						
Digit Span	512	7.11 (2.86)	7.22 (2.26)	0.04	−[0.16, 0.23]	.72
Cognitive ability						
IQ	513	82.72 (13.81)	81.46 (12.17)	−0.09	−[0.30, 0.11]	.37
Attention set-shifting						
Total errors—set-shifting task	271	43.63 (21.50)	46.42 (19.34)	0.13	−[0.14, 0.40]	.34
ED shift errors	271	17.55 (10.12)	19.28 (9.54)	0.17	−[0.10, 0.44]	.21
Motivational deficits						
Quality of decision making	289	0.75 (0.20)	0.78 (0.17)	0.17	−[0.10, 0.43]	.22
Delay aversion	203	0.56 (0.21)	0.62 (0.19)	0.29	−[0.02, 0.60]	.07
Risk taking	287	0.54 (0.17)	0.55 (0.15)	0.07	−[0.20, 0.33]	.63
Risk adjustment	287	0.31 (0.90)	0.27 (0.87)	−0.05	−[0.32, 0.22]	.72

*Note.* CI = confidence interval; ED = extra dimensional shift stage (Stage 8).

a Neurocognitive outcome variables in the unadjusted model are based on standardized scores.

**Table 4.** Associations Between Parent Depression (Mother/Father) and Child Neurocognitive Performance Adjusting for Covariates (Low Parent Education Status, Low Social Class, Child Age, Child IQ, ADHD, and Conduct Symptom Severity).

Neurocognitive outcome	Model 1(Low parent education, low social class & age)				Model 2 + (IQ)			Model 3 +(ADHD & CD severity)	
	*B*	(95% CI)	*p*	*B*	(95% CI)	*p*	*B*	(95% CI)	*p*
Working memory									
Digit Span^a^	0.13	−[0.08, 0.34]	.22				0. 16	[0.06, 0.38]	.15
Cognitive ability									
IQ	0.01	−[0.20, 0.23]	.91				0.05	−[0.16, 0.27]	.62
Attention set-shifting									
Total errors—set-shifting task	0.15	−[0.12, 0.43]	.28	0.11	−[0.16, 0.39]	.43	0.14	−[0.14, 0.43]	.31
ED shift errors	0.20	−[0.09, 0.48]	.18	0.14	−[0.15, 0.43]	.34	0.16	−[0.13, 0.46]	.27
Motivational deficits									
Quality of decision making	0.28	[0.01, 0.54]	.04	0.26	−[0.02, 0.53]	.07	0.25	−[0.03, 0.53]	.08
Delay aversion	0.20	−[0.12, 0.52]	.22	0.17	−[0.15, 0.49]	.30	0.18	−[0.14, 0.52]	.27
Risk taking	0.16	−[0.10, 0.42]	.24	0.17	−[0.09, 0.43]	.18	0.16	−[0.10, 0.44]	.23
Risk adjustment	0.04	−[0.24, 0.32]	.80	0.02	−[0.27, 0.30]	.91	0.06	−[0.23, 0.35]	.67

*Note.* Neurocognitive outcome variables in all models are based on standardized scores. CD = conduct disorder; CI = confidence interval; ED = extra dimensional shift stage (Stage 8).

a Digit Span subtest is included as part of the full scale IQ estimate; therefore, this was not controlled for.

In view of the high proportion of missing information on fathers, we examined whether there were differences between children with complete parent information and those without. There were no differences in mean scores of performance on the neurocognitive tasks between children with and without complete parent information. (Results available from first author)

## Discussion

This study aimed to build upon previous findings that parental ADHD and depression symptom presence are associated with a clinically more severe presentation of ADHD as defined by reported symptoms (Agha et al., 2013, 2017; Chronis et al., 2003; Humphreys et al., 2012; López Seco et al., 2015; Pressman et al., 2006; Segenreich et al., 2014). As previously described (Agha et al., 2013, 2017), rates of parental psychopathology were high; 33% of children had a parent with ADHD symptom presence and 24% had a parent with depression symptom presence. As previously published, parental psychopathology was associated with offspring ADHD symptom severity and the presence of comorbid CD (Agha et al., 2013). We were interested in whether these previously described associations between parental psychopathology and offspring ADHD clinical severity (Agha et al., 2013, 2017) extended to alternative, more objective, measures of offspring difficulty, that is, impaired neurocognitive performance.

The findings indicated that children who had a parent with ADHD symptom presence performed more poorly in measures of working memory (the digit span task) and set-shifting ability (number of errors in the ED shift stage and completion of stage 8/9 of the set shifting task). The associations remained even after controlling for ADHD and CD severity. However, no differences were found in the domains of general cognitive ability (full scale IQ) or motivational deficits in decision making (measured by the Cambridge Gambling Task). These findings for set-shifting ability are similar to those reported by Seidman and colleagues (Seidman et al.,1995, 1997) where family history of ADHD (in first degree relatives including siblings) was found to predict impairment in the Wisconsin card sorting tasks (WCST) which is akin to the IE/ED set-shifting task.

Our finding of association between parent ADHD symptom presence and poorer offspring working memory differ from those of Thissen and colleagues who found no association in their study of 259 adolescents with ADHD (Thissen, Rommelse, Hoekstra, et al., 2014). This difference may be due to the slightly different task measures used between these two studies and the different ages (mean age: 17.3 years vs. 10.78 in this study) of the individuals studied and smaller sample sizes, which highlights the need to take such task and sample characteristics into account in such studies. Although cognitive tasks are perhaps more objective than subjective reports, one problem is that there is no single gold standard method for assessing specific neurocognitive constructs.

Nonetheless, there has been recent evidence which found composite ADHD molecular genetic risk scores are associated with lower IQ and working memory performance as well as ADHD symptom levels in children in the general population (Martin, Hamshere, Stergiakouli, O’Donovan, & Thapar, 2015). This suggests that the genetic risk for ADHD is also relevant to lower IQ and working memory abilities; however, the present study focuses on variation within ADHD patients only and all of them are impaired. Taken together, the findings indicate that association between parent ADHD symptom presence and lower performance in working memory might be an indicator of higher genetic risk.

In contrast to the findings for children with parents who have ADHD, no associations were found between parent depression status and offspring neurocognitive performance. These findings support and extend those of a much smaller pilot study (Park et al., 2014) which found no evidence of differences in working memory, cognitive ability, or set-shifting for children with ADHD between those with and without a parent with a history of mood disorder. Motivational decision making was not investigated by this group. It is important to note here that the measure of parent ADHD in the present study perhaps indexes more longstanding symptoms from childhood to now, whereas parent depression is only measured currently. This perhaps might explain why associations were found with parent ADHD and not parental depression; the parent ADHD measure is indexing more severe psychopathology, and depression can be a relapsing and remitting disorder unlike ADHD.

This study is one of the first studies to investigate the links between parent psychopathology and variation in offspring neurocognitive performance in a large clinical sample of children with ADHD. It includes the analysis of both parent ADHD and depression symptom presence within the same sample and explores associations with variation in offspring neurocognitive functions implicated as being affected in ADHD including delay aversion and decision making, which have not been examined previously. Overall, the results of this study highlight that children with ADHD who already have neurocognitive deficits relative to the general population, and who have a parent with ADHD symptom presence may experience even greater neurocognitive problems, which underscores the importance of considering parent mental health during clinical assessment. Parent mental health problems appear to be linked to both cognitive as well as clinical indices of ADHD severity in clinic children; this is in the context of elevated social adversity that commonly accompanies parent psychopathology. Mechanisms that account for these cross-generational links likely include genetic, biological, and social ones.

As with any investigation, this study should be considered in view of certain limitations. Measures of parent ADHD were based on self-report and retrospective recall of childhood ADHD symptoms. Evidence from previous studies however has suggested that adults can give a reasonable account of their own childhood and current symptoms (Murphy & Schachar, 2000), although there are other studies which suggest otherwise (Moffitt et al., 2010; Moffitt et al., 2015). There was also unfortunately no measure of impairment or pervasiveness for parent ADHD. Depression status for parents in this study was obtained using a cut point on a widely used, validated scale, the HADS which was initially developed for screening purposes and therefore does not represent a *DSM-5* diagnosis of major depressive disorder. However, the HADS has been reported to have good validity and performs well in predicting caseness of anxiety disorder and depression in both psychiatric and primary care patients as well as the general population (Bjelland et al., 2002). Unfortunately, there was no measure of parental IQ and parents were not assessed on the same neurocognitive tasks as their children. However, we controlled for parent education in the analyses as a proxy measure of parent IQ, and associations between parent ADHD symptom presence and child performance on neurocognitive tasks remained unchanged. Although participants in this sample have performed more poorly on all neurocognitive tasks compared with published population norms, it is important to note that this may partly be due to the IQ of participants in this study being below average, and therefore findings are perhaps more relevant to a clinical sample of ADHD.

Thissen and colleagues found that there may also be different influences of mother and father ADHD on the child’s neurocognitive task performance (inhibition; Thissen, Rommelse, Altink, et al., 2014). However, the results on parent gender differences are inconsistent as there are other studies that failed to show any differences between mother and father psychopathology in relation to offspring neurocognitive impairment (Crosbie & Schachar, 2001; Goos et al., 2009). In this study, we used a combined parental measure of psychopathology, that is, for either mother or father to have symptom presence of ADHD or depression. This was decided *a priori* to increase statistical power and because there was no evidence of assortative mating for parent ADHD (χ2 = 0.63 (1), *p* = .43) and parent depression (χ^2^ = 1.68 (1), *p* = .19) status. However, sensitivity analyses were also conducted to examine association for mother and father psychopathology separately, and weak evidence of associations was found for working memory (mother ADHD: *B* = −0.18, 95% CI [−0.39, −0.03], *p* = .09, father ADHD: *B* = −0.25, 95% CI [−0.51, 0.003], *p* = .05).

A large proportion of individuals ascertained in this sample were from single parent families (mostly mothers), typical of referrals to many child and adolescent mental health services in the United Kingdom where health care is free of charge so those from high-risk backgrounds are well represented in clinics. Therefore, information on a substantial number of fathers was missing, and the rates of parent ADHD and depression likely will have been underestimated. Sensitivity analyses were conducted in the sample, and we found that there were no differences in associations for children with data available from both parents and those from single-parent families.

Finally, the findings of this study are in need of replication considering that we did not perform any corrections for multiple testing. The outcomes for each task in this study are correlated with each other and some have suggested that traditional methods of correcting for multiple testing, such as the Bonferroni method, would be overly conservative in situations like this (Perneger, 1998). This was an exploratory study, and findings help add potential insight into how parent ADHD is related to some aspects of offspring neurocognitive performance, which is another important manifestation of the ADHD phenotype.

## Clinical Relevance

There have been a few advances in the development of intervention strategies for children with ADHD that target neuropsychological impairments (Halperin et al., 2013; Tamm, Nakonezny, & Hughes, 2014; Tarver, Daley, & Sayal, 2014). These interventions encourage parental involvement in adopting strategies and techniques aimed at improving aspects of executive functioning deficits and abnormal reward processing (Tamm et al., 2014). Examples include play and exercise activities to develop inhibitory control, (e.g., Simon says games), working memory, and altering reward processing (immediate parental reinforcement; Halperin et al., 2013). Preliminary evidence from this has shown improvements in executive functioning performance and ADHD severity post treatment (Halperin et al., 2013; Tamm et al., 2014). However, interventions such as these depend heavily on parental involvement, and optimal engagement from parents depends very much on many factors including parent mental health (Tarver et al., 2014). Therefore, understanding the association between parent psychopathology and neurocognitive deficits in children with ADHD is important and relevant for the development of intervention and treatment plans specifically tailored for subgroups of high-risk children.

In conclusion, the results of this study suggest that parent ADHD symptom presence is related to poorer performance in set-shifting and working memory in their offspring with ADHD, but that parent depression symptom presence is not associated with impaired offspring neurocognitive functioning measured in this study. This further extends findings that parental ADHD is associated with offspring ADHD severity and again highlights the importance of considering parental mental health when assessing child ADHD.
